# The Therapeutic Employment of the Fumes of Opium

**Published:** 1869-03-01

**Authors:** 


### CHICAGO MEDICAL JOURNAL.

VOL. XXVI.—MARCH 1, 1869.—No. 5.

### TRANSLATIONS.

### *The Therapeutic Employment of the Fumes of Opium.*

Doctor Armand, a military medical officer read, recently, to the Academy of Medicine, Paris, a paper under this title, expressing the belief that, medically speaking, the fumes of opium might be very useful. He refers to the declaration of Sydenham, that he would rather renounce the practice of medicine than the employment of opium. Not doubting that the illustrious English physician would have utilized the fumes of opium in the Chinese method had he been acquainted with it, the author continues: Indeed! after several years of experimentation, during which we employed the fumes of opium as a medical agent, we will not defer longer the publication of this very simple, inoffensive and very fruitful means to be directed against a series of chronic and neuralgic affections, especially of the chest.

But first, how should opium be smoked? With a special pipe, of which we give a description.

The stem of an opium pipe resembles a flute, having for its only lateral opening that of the mouth-piece. It is therefore a tube closed at one end, near which there is pierced, laterally, a circular opening, to which is adapted the neck of a pipe. This pipe has no resemblance to a tobacco pipe. It is formed of a sprinkling nozzle, having only a very small hole of scarcely a millimetre (.039 in.) in diameter in the middle of its slightly convex surface.

The pipe of baked clay of six or seven centimetres (2.24 to 2.73 in.) in diameter and four in depth, is hollow interiorly, and terminates in a neck of one centimetre (.39 in.) in diameter. These dimensions of the pipe, as well as those of the large tube, of at least fifty centimetres (19.4 in.) in length are determined in order to cool the smoke.

In order to charge the pipe, one or two grains of extract of opium should be taken upon the point of a steel needle.

This piece of the extract is to be applied to the flame of a lamp, in order to put it in a state of ebullition, and to give to it a sufficient degree of consistence to permit it to remain in a state of inflation.

This piece of prepared opium is then placed, very carefully, near the little central hole of the pipe, but without obstructing it, which is effected by means of the point of the needle, which is always held in the hand. This done, the central portion of the pipe is exposed to the flame of a lamp or candle. The opium assumes the state of ebullition, ignites, and this is the moment for the aspiration of the smoke, by a deep pulmonary inspiration.

The pipe is then withdrawn from the flame, the smoke retained for an instant in the bronchi, and thence returned by an expiration more or less prolonged, either through the mouth or through the nostrils. The point of the needle is then freshly inserted in order to maintain the opening of the central hole; the pipe replaced in the flame, and a new aspiration made. If in boiling, the opium which remains flows away from the centre, which happens if it is not sufficiently ignited, the point of the needle serves to replace it before it has cooled. After the fourth or fifth aspiration all the opium will have been burned. The charred residuum may be removed with the needle, using it as a scraper, and it must be renewed if it is desired to continue the aspirations or rather the inspirations.

Indeed, a simple aspiration with the mouth (buccale), as with the tobacco pipe, would not suffice to burn the opium; it would scarcely suffice to introduce a little smoke into the mouth, which would avail nothing. The true method of habitual smokers, is to make not simply a buccal but a pulmonary aspiration; in a word a strong inspiration as prolonged as possible, filling the bronchi with air loaded with the fumes of opium. The smoke must then be inspired to the full capacity of the lungs, and this is the indispensable condition not only to burn the opium but to receive its effect.

It is necessary to observe carefully that the opium must not be ignited by the flame of a smoky lamp. Moreover, our oil lamps are worthless for this purpose, on account of the grease with which they contaminate the opium and load its fumes. The alcohol lamp irritates the throat and the bronchi; carburetted hydrogen gas as well as petroleum are mephitic.

At Singhapour the virgin oil of cocoa is used in the lamps for burning opium. By analogy we have tried the oil of almonds, but even, this was too smoky, and we now prefer the flame of a wax candle simply.

Among other physiological and therapeutical effects of the fumes of opium, we will mention the following: a person in good health who smokes opium for the first time, finds the smoke agreeable and pleasant to the taste and smell. From the first pipe he is a smoker; and except while making a very violent inspiration, the smoke of opium never occasions cough. This fact is very important in a therapeutic point of view.

Now, how many grains of opium can an adult smoker burn without being perceptibly affected? At the beginning of our personal experiments we investigated this point of the question frequently, and can affirm that before having burned fifty centigrammes (about 10 grs.) of opium, we were never affected sensibly. Beyond that quantity there was general heat, and during sleep there were dreams anything but agreeable, and approaching to nightmare.

Have we, therefore, become an opium smoker? Assuredly not; the smoke of opium, though agreeable, had not for us irresistible attractions. The idea of smoking opium does not recur to us perhaps three times in a year.

There is no danger of being drawn beyond the limits of prudence and necessity in smoking opium. Without meaning to dispute that excess is always injurious, we assert that the effect of smoking opium upon the great smokers of Indo-China has been greatly exaggerated.

By keeping within prudent limits, that is to say, smoking from one to ten grains of opium during twenty-four hours, there is obtained a very salutary sedative, and beneficial medical effect in the following cases:

In the first rank we cite cases of chronic bronchitis and laryngitis, not excepting the acute forms of these affections; pertussis in patients capable of smoking the opium pipe; cases of asthma, of anguina pectoris, and of nervous palpitations, of gastralia and of enteralgia.

In the second place, neuralgia, facial, dental, sub-orbital and hemicranial; muscular and articular rheumatism; for it should be observed especially that beside the sedative action of the smoke of opium, there is likewise, from large doses the general calorification and sudation or diaphoresis, which the practitioner may excite and employ according to the clinical indications. We will conclude by replying, in advance, to an objection or rather an observation which we anticipate:

It will be asked, why not substitute for the opium pipe, opiated cigarettes? At one time we were thus deluded. We sought and discovered a substance burning readily and readily impregnated with the watery solution of the extract of opium, and capable of being made into opiated cigarettes composed as follows :

Take ; pulverized flower of the linden in sufficient quantity to make twenty cigarettes—

Impregnate these flowers with an aqueous solution of one gramme of extract of opium ; let them dry in the air, moderately, and make of them twenty cigarettes, containing one grain (five centigrammes) of extract of opium. When dried sufficiently let them be smoked in the usual manner.

The result has not answered our expectations ; although mild the smoke of the opiated linden flowers, especially when mingled with that of the paper, which is always acrid and irritating, does not permit pulmonary aspirations to be made.

It becomes necessary to restrict the aspirations to the mouth, and it is with difficulty that the taste of the opium can be perceived in the smoke, which, in fact, is largely, if not totally evaporated before reaching the mouth.

What has been said of opium cigarettes may be said of all medicated cigarettes, and it is our conviction that every medicinal substance used by inhaling its smoke, should be burned in the state of extract, as the extract of opium, in an opium pipe, whose name we will some day change for that of *pipe for smoking medicinal extracts.*

